# Supplementary material for: Application of polygenic scores to a deeply phenotyped sample enriched for substance use disorders reveals extensive pleiotropy with psychiatric and somatic traits
Source: Neuropsychopharmacology. 2024 Jul 23;49(13):1958–67. doi: 10.1038/s41386-024-01922-2 (PMC11480112; doi:10.1038/s41386-024-01922-2)

Supplementary Figure 1: PGS_{MDD} covarying for MDD

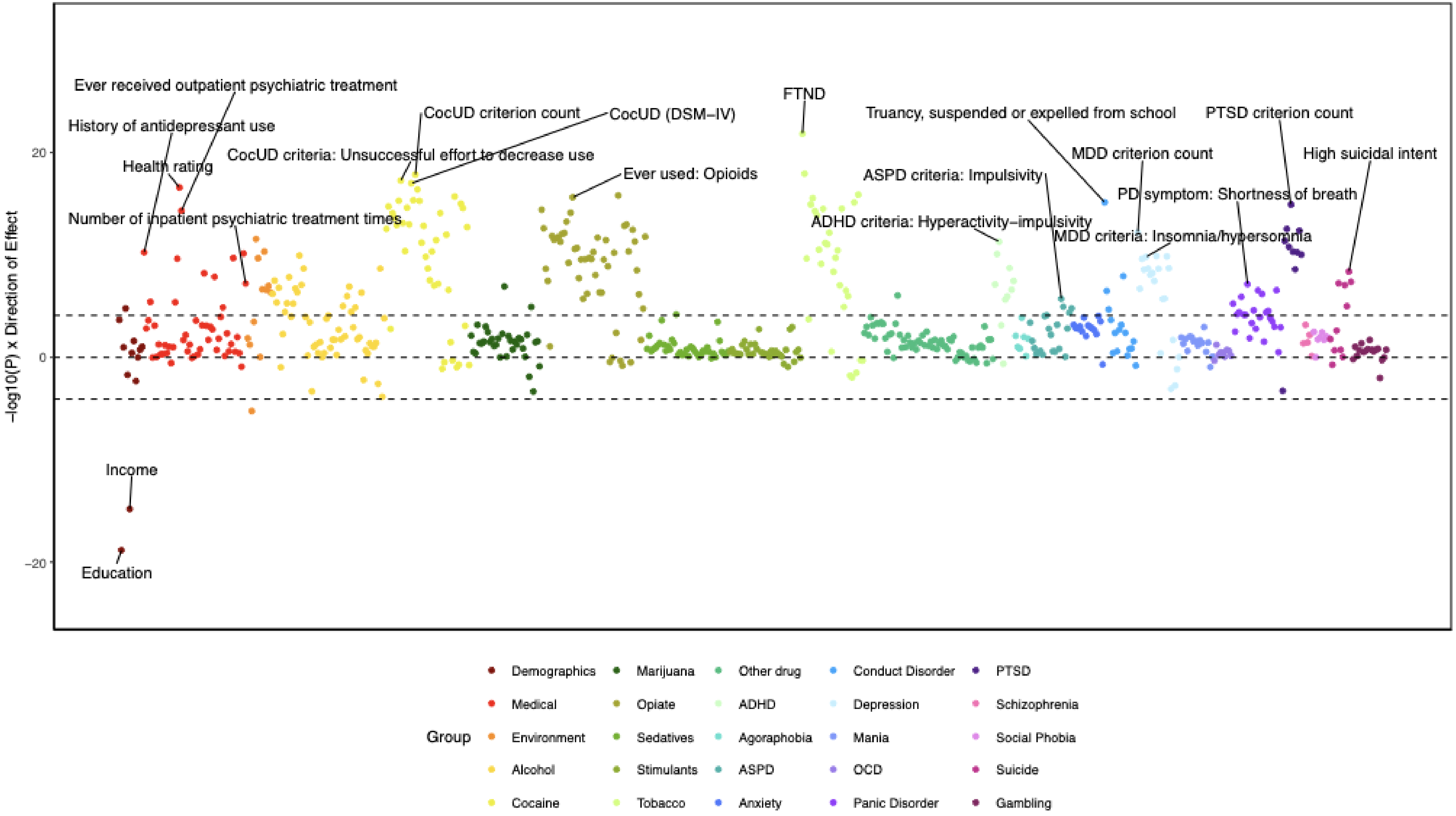

Supplementary Figure 2: PGS_{GAD} covarying for GAD

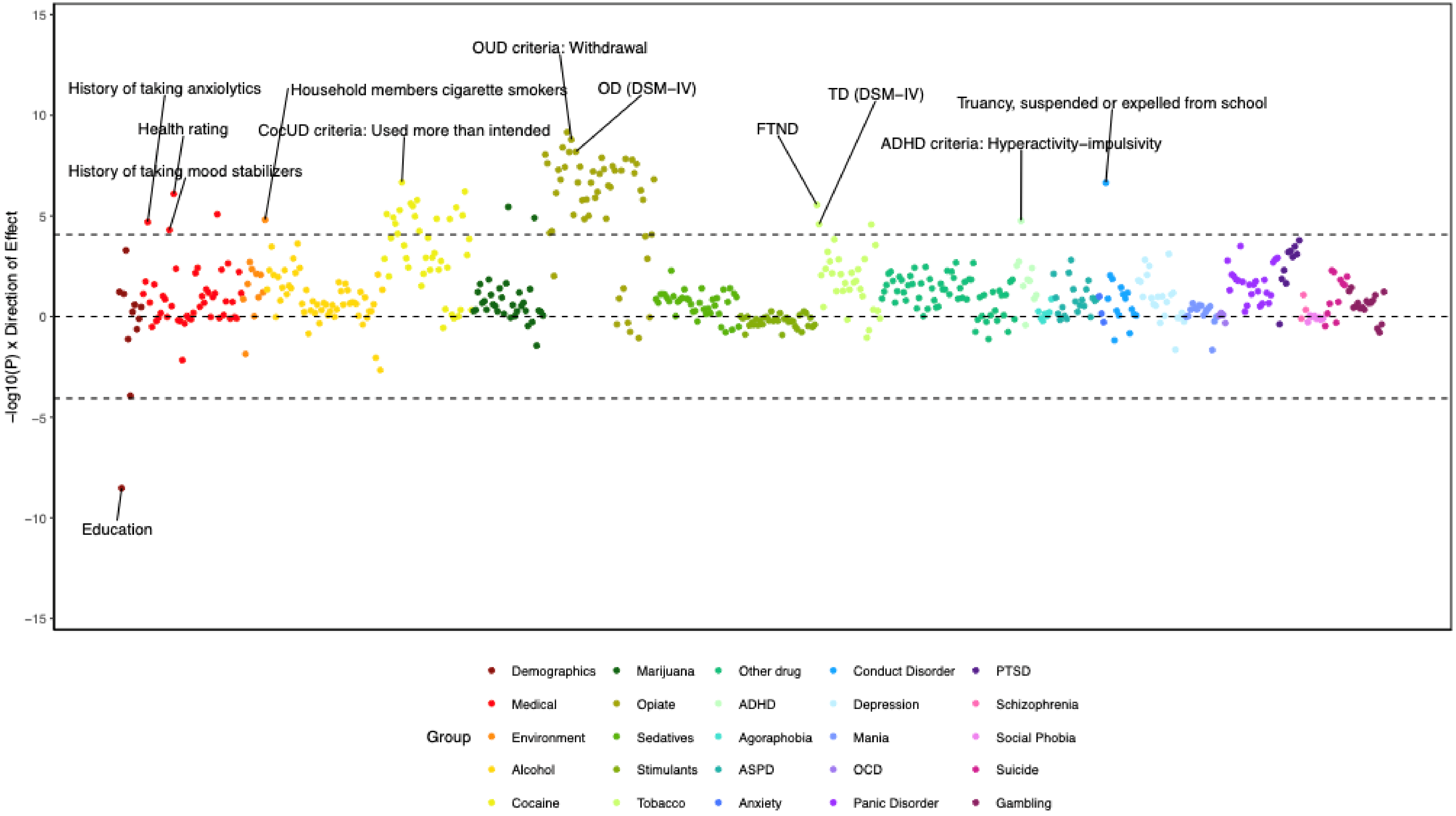

Supplementary Figure 3: PGS_{PTSD} covarying for PTSD

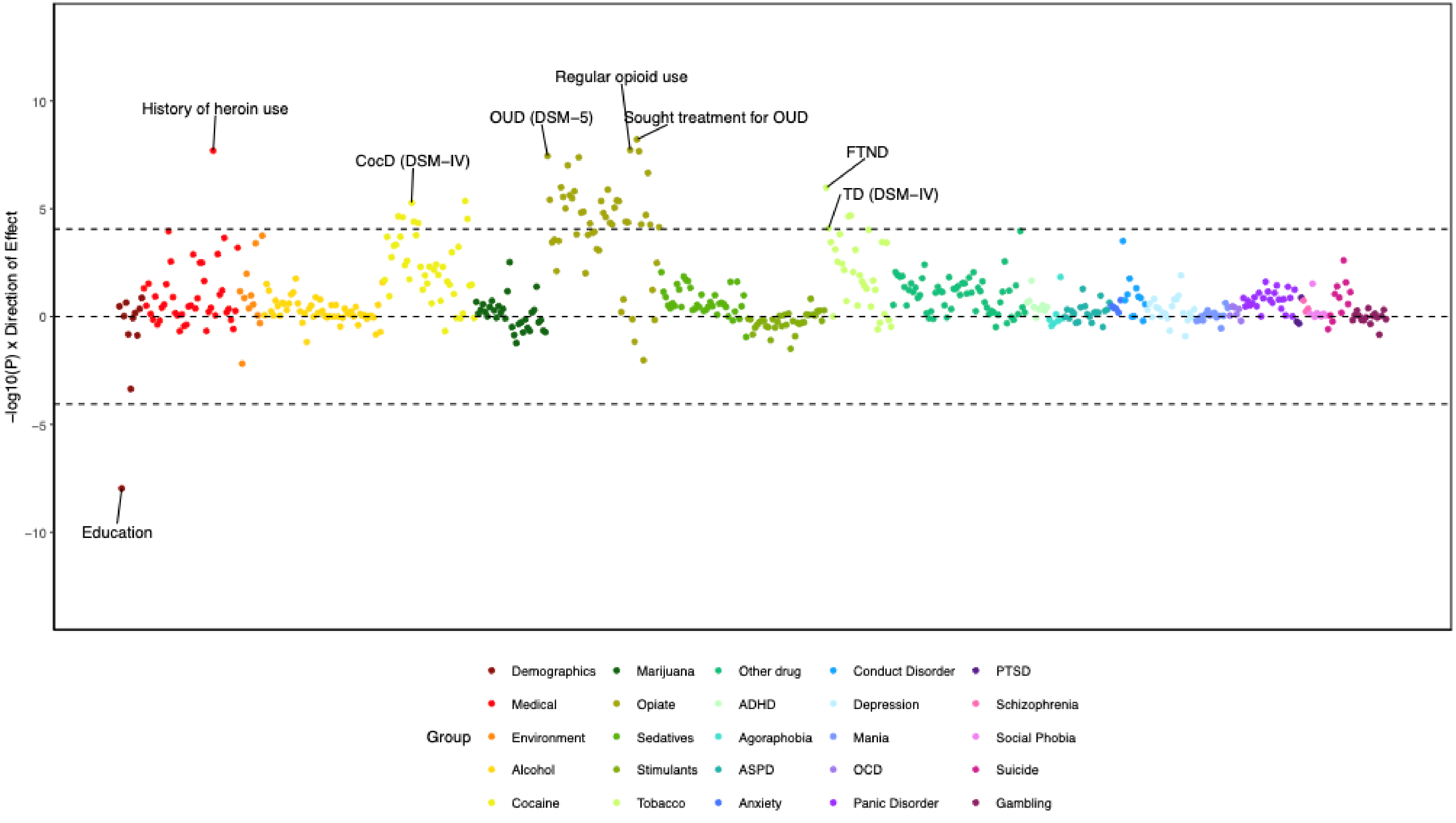

Supplementary Figure 4: PGS_{BMI} covarying for BMI

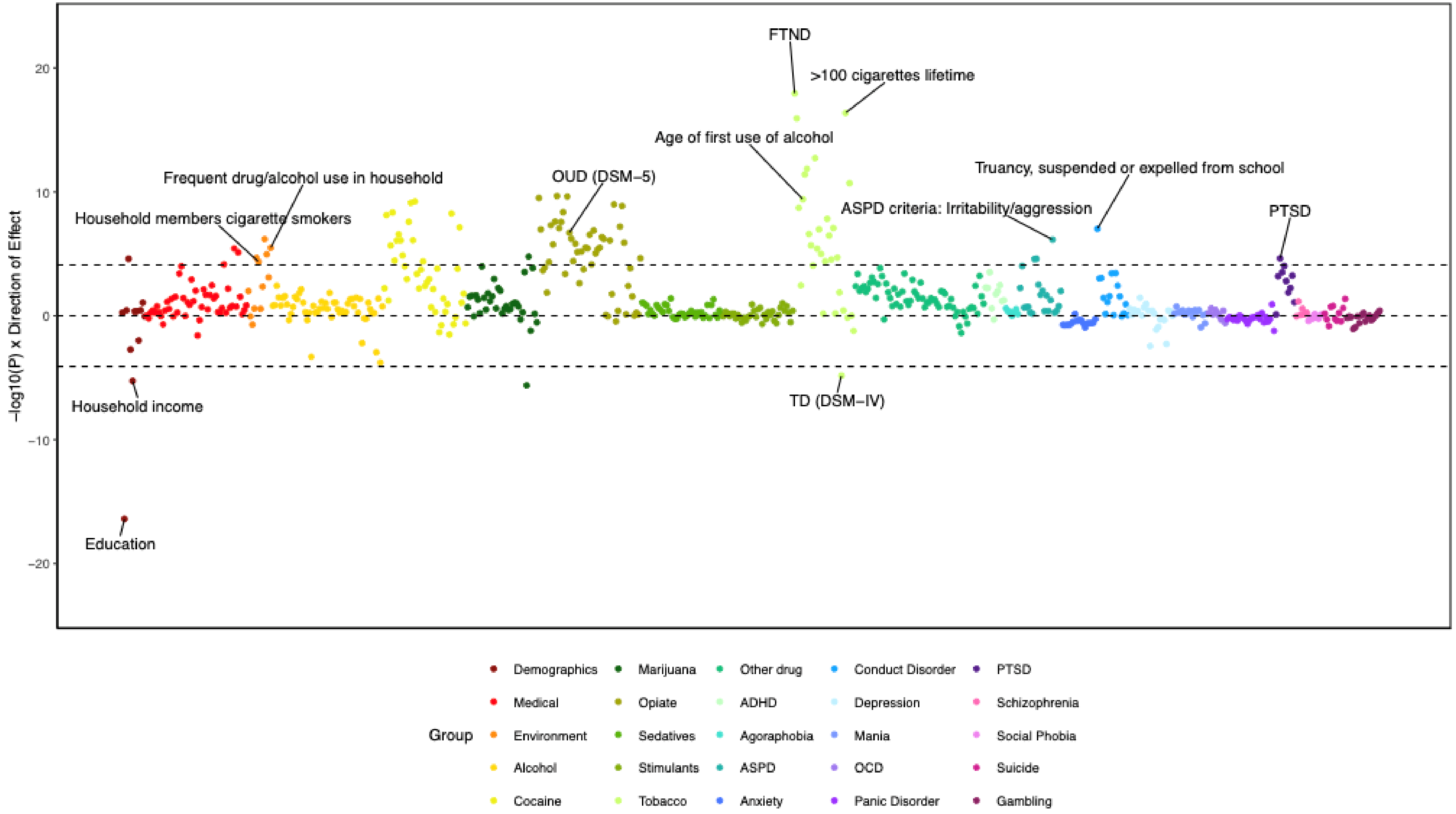

Supplementary Figure 5: PGS_{CAD} covarying for CAD

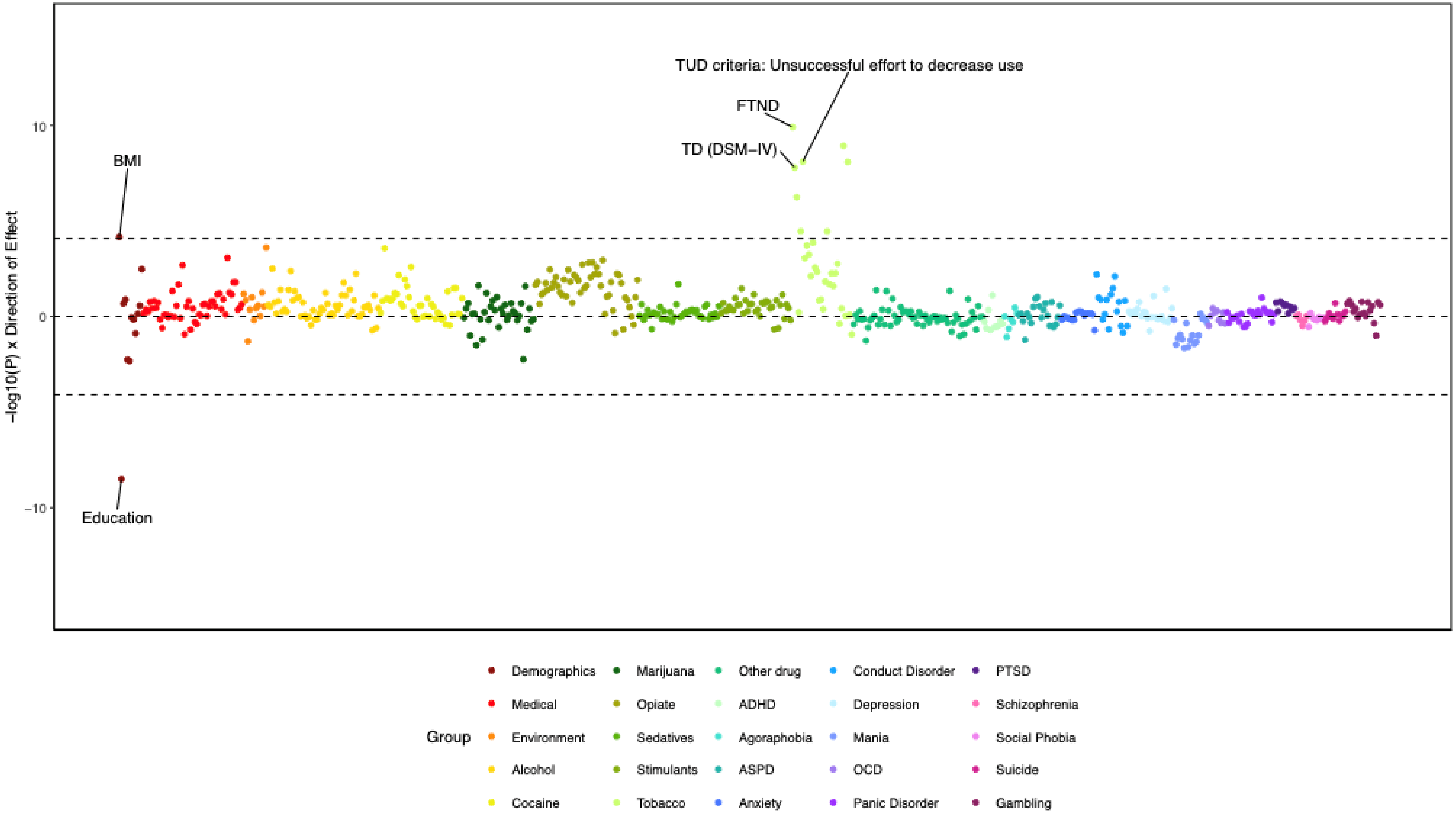

Supplementary Figure 6: PGS_{T2D} covarying for T2D

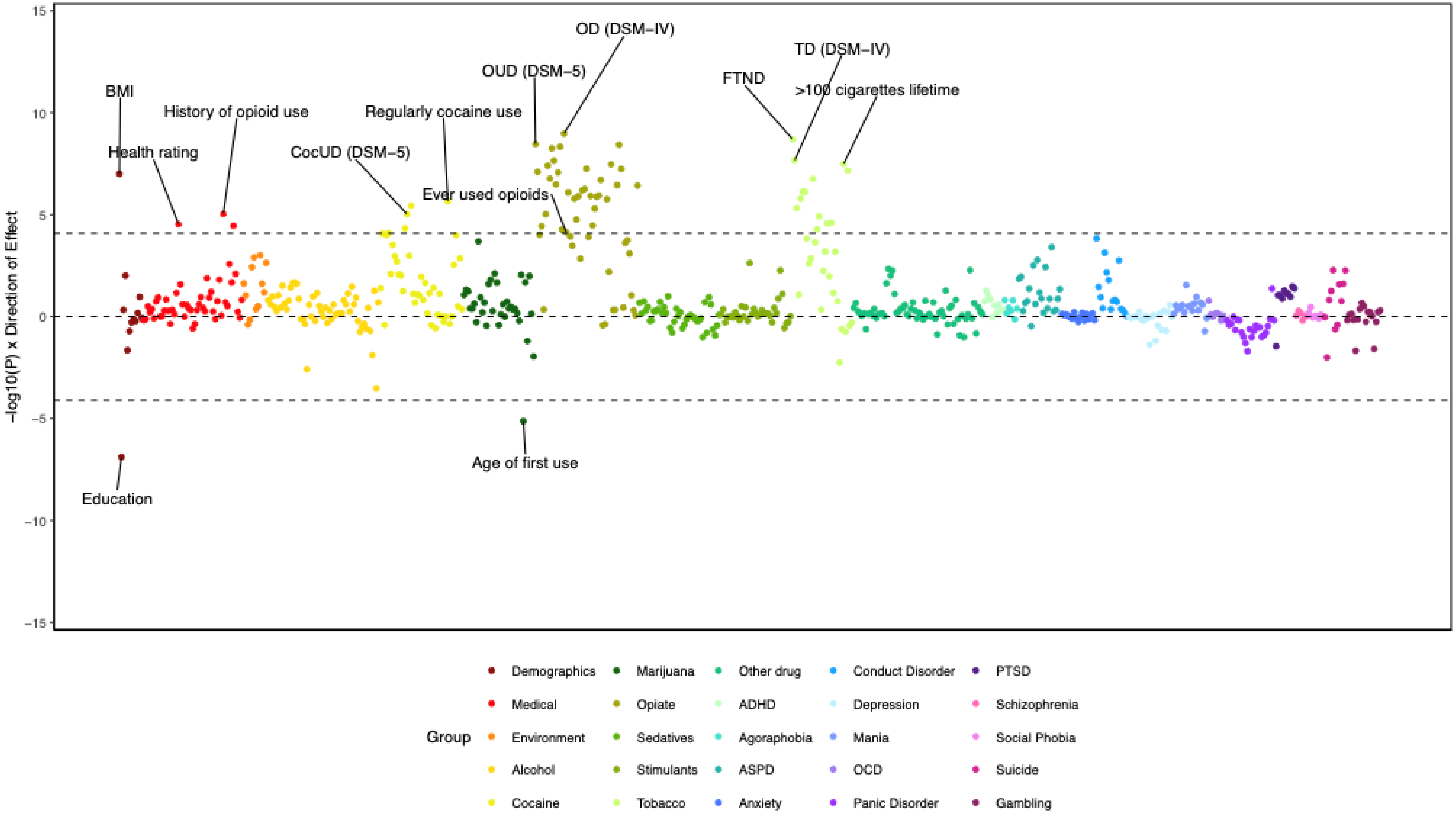

Supplement: Supplementary file 1 — Supplementary Figures [file 41386_2024_1922_MOESM1_ESM.pdf]
